# Protective Role of Hepcidin in Polymicrobial Sepsis and Acute Kidney Injury

**DOI:** 10.3389/fphar.2019.00615

**Published:** 2019-06-06

**Authors:** Yogesh Scindia, Ewa Wlazlo, Joseph Leeds, Valentina Loi, Jonathan Ledesma, Sylvia Cechova, Elizabeth Ghias, Sundararaman Swaminathan

**Affiliations:** Division of Nephrology, University of Virginia Health System, Charlottesville, VA, United States

**Keywords:** acute kidney injury, endotoxemia, sepsis, spleen, macrophage, hepcidin

## Abstract

**Background:** Acute kidney injury (AKI) portends worse prognosis following sepsis, with limited available interventions. Host iron acquisition by pathogens and systemic inflammatory response are key events in the pathogenesis of sepsis. In sepsis, hepcidin induces iron sequestration to limit iron availability to pathogens. Hepcidin is also known to limit inflammation. Since its role in pathophysiology of sepsis-associated AKI is unknown, we investigated the effect of exogenous hepcidin in endotoxin- and peritonitis-induced pathology and AKI.

**Methods:** C57BL/6 mice were treated with saline or 50–100 µg of hepcidin, pre- and post-LPS injection, or cecal ligation and puncture (CLP, model of peritonitis). Splenectomized mice were challenged with LPS, with and without hepcidin. Mice were euthanized at 24 h after LPS injection and at different time points after CLP. Systemic inflammation and renal injury markers were assessed. Direct effect of hepcidin on renal tubular and endothelial cells was evaluated using endotoxin-induced cytotoxic serum. Role of heavy chain ferritin (H-ferritin) in mediating hepcidin-induced anti-inflammatory effect on LPS stimulated macrophages was evaluated with siRNA studies.

**Results:** Twenty-four hours pretreatment with hepcidin significantly reduced LPS-induced AKI. Hepcidin ameliorated LPS-induced increase in serum TNFα and renal Cox-2, and prevented loss in PGC1α and cytochrome c oxidase activity. This was associated with reduced glomerular injury and preserved mitochondrial structure. Hepcidin did not exert direct protection on the renal parenchymal cells but reduced endotoxin-induced serum cytotoxicity to mitigate renal injury. Splenectomy reduced LPS-induced early inflammation and AKI, independent of hepcidin, indicating the importance of systemic inflammation. Higher splenic H-ferritin in hepcidin-treated animals was associated with reduced splenocytes apoptosis and inflammation. Hepcidin reduced LPS-induced IL-6 secretion in macrophages in H-ferritin dependent manner. Hepcidin significantly reduced CLP-induced AKI, and mortality (20% hepcidin treated vs 80% PBS treated). Importantly hepcidin reduced bacteremia and AKI even when administered after onset of sepsis.

**Conclusion:** We demonstrate a protective role of hepcidin in endotoxin- and peritonitis-induced pathologies and AKI, exerted primarily through its anti-inflammatory effects, and antibacterial property. Macrophage H-ferritin plays an important role in hepcidin-mediated protection against endotoxin-induced inflammation. We uncover a novel prophylactic and therapeutic role of hepcidin in sepsis-associated bacteremia, AKI, and mortality.

## Introduction

Sepsis is a common trigger for acute kidney injury (AKI) in critically ill patients representing approximately 50% of all AKI cases in intensive care units (Uchino et al., 2005; Emlet et al., 2015). Sepsis-associated AKI (SA-AKI) portends a higher risk of mortality (Bagshaw et al., 2007; Martensson and Bellomo, 2015). The pathogenesis of SA-AKI is complex and involves systemic cytokine storm, mitochondrial dysfunction, tubular epithelial cell injury, and endothelial dysfunction (Havasi and Borkan, 2011; Zarjou and Agarwal, 2011; Parikh et al., 2015). However, limited understanding of pathophysiologic mechanisms has precluded the development of effective therapies. It is therefore necessary to explore new mechanisms and pathways to prevent the development of SA-AKI. Animal studies have shown that lipopolysaccharide (LPS) induces systemic inflammatory response syndrome (SIRS) with AKI and hence is widely used for mimicking sepsis and drug development (Remick et al., 2000; Knotek et al., 2001; Bhargava et al., 2013).

Iron and related proteins have been implicated in SA-AKI (Martins et al., 2016; Weiss and Carver, 2017). Zager et al. (2005) demonstrated that intravenous but not intramuscular injection of iron compounds accentuated LPS-induced TNF-α production and AKI. This observation indicates the relevance of hepcidin (hepatic antimicrobial peptide or Hamp)-induced hypoferremia observed as an acute phase response following LPS injection (Bertini et al., 1989; Kemna et al., 2005; Armitage et al., 2011; Deschemin and Vaulont, 2013; Rodriguez et al., 2014). Hamp-mediated hypoferremia functions as a host defense mechanism that has evolved to restrict iron availability for pathogen growth (Ganz, 2009; Drakesmith and Prentice, 2012). In a cholestasis-induced liver injury model superimposed with sepsis, pretreatment with Hamp mitigated liver injury and reduced systemic IL-1, TNFα, and MCP-1 (Huang et al., 2012). Similarly, Hamp reduced TNFα, IL-1, and IL-6 in LPS stimulated macrophages (Rajanbabu et al., 2010; Rajanbabu and Chen, 2011).

In human sepsis-induced AKI, there is a complex interplay between positive and negative regulation of Hamp with consequent alteration in distribution of iron (Schaalan and Mohamed, 2016). Kidney transplant recipients with high ferritin levels exhibit an increased incidence of post-transplant infection and are associated with elevated baseline serum hepcidin-25 levels (Fernandez-Ruiz et al., 2018). Furthermore, neutrophil gelatinase-associated lipocalin (NGAL)/hepcidin ratio is strongly associated with severe AKI than the single biomarkers alone (Martensson et al., 2015). Although these studies highlight changes in Hamp following sepsis-associated AKI, the role of Hamp or iron transport in the pathophysiology of SA-AKI has not been investigated in detail. In this study, we examined whether exogenous Hamp can protect against LPS- and CLP-induced pathology and AKI and explored the associated mechanisms.

## Methods

### Mice and Systemic Inflammatory Response Syndrome Induction

All experiments were performed in accordance with the National Institutes of Health and Institutional Animal Care and Use Guidelines. The Animal Care and Use Committee of the University of Virginia approved all procedures and protocols. Male, 8- to 10-week-old C57BL/6 mice (The Jackson Laboratory, Bar Harbor, ME) were used throughout this study. Seven-week-old splenectomized mice were purchased from Jackson Laboratory and used for experiments 1 week later. LPS (*Escherichia coli* 0111: B4, Sigma-Aldrich, Milwaukee, WI, USA) was freshly dissolved in sterile phosphate buffered saline (PBS). Mice were injected intraperitoneally with LPS (6.5 mg/kg) and followed for 24 h. Animals were injected with PBS or Hamp (50 µg/mouse, i.p.; Peptide International) 2, 8, 12, or 24 h prior to LPS injection. In some experiments Hamp was injected 3 h after LPS, and mice were followed for 24 h. Blood and tissues were collected as previously described (Scindia et al., 2015).

### Mouse Model of Polymicrobial Sepsis

Male, 8- to 9-week-old C57BL/6 mice (The Jackson Laboratory, Bar Harbor, ME) were injected with PBS or Hamp (100 µg), as indicated in **Figure 7A**. Mice were anesthetized using a mixture of ketamine/xylazine and a 1.5-cm midline incision was made along the abdominal wall. The cecum was exposed and ligated immediately below the ileocecal valve. A 22-g needle was used to make a through and through puncture close to the ligated ileocecal valve, to extrude a small amount of fecal matter. The cecum was replaced into the abdominal cavity and the wound was closed in two layers with a running 5.0 silk suture. Mice were volume resuscitated with 0.5 ml normal saline subcutaneously. Care was taken to maintain similar timing between surgery and euthanasia in the two animal groups by randomizing mice for surgery. All mice were injected buprenorphine, every 12 h for the first 48 h to relieve pain. Animals were euthanized 4.5 h, 9 h or 6 days later. In some experiments, mice were injected with 100 µg Hamp, 2.5 h after induction of CLP.

### Renal Function

Blood urea nitrogen was determined using a commercial detection kit (Arbor Assays). Plasma creatinine was determined using a modified Jaffe colorimetric assay (Gigliotti et al., 2013).

### Non-Heme Iron Assay

Kidney non-heme iron (µg iron/gm tissue) was measured as described previously. Briefly, accurately weighed tissue sections were finely cut and incubated with 3 M HCl/0.61 M trichloroacetic acid mixture for 20 h at 65°C. After cooling, the acid extract was spun at 12,000 rpm, and 0.1 ml of the supernatant was mixed with 1.86 mM bathophenanthroline sulfonate and 143 mM thioglycolic acid for 20 min, and optical density (OD) was measured at 535 nm. A standard curve was generated using an iron standard solution (Ricca Iron AA standard) against water as the blank.

### Immunofluorescence

Three-micron, PLP-fixed kidney and spleen sections were used for the immunofluorescence detection of ferroportin, neutrophils (7/4 antigen), CD11b+ve cells, and F4/80 macrophages. Briefly, tissue sections were air dried and incubated with 0.3% Triton X-100/10% horse serum in PBS for 30 min. After washing in PBS, anti-CD16/32 antibody was added to block F_C_ receptors (2.4g2 eBioscience). This was followed by 2-h incubation with ferroportin antibody (G-16, goat polyclonal, Santa Cruz Biotech). Ferroportin was detected using a FITC-labeled Donkey anti-goat secondary antibody. Sections were incubated with FITC-labeled 7/4 (Cederline, 1:25), PE-labeled CD11b (M1/70, eBioscience, 1:30), and APC-labeled F4/80 (BM-8, eBioscience, 1:30) in 10% horse serum/PBS for 1.5 h. The sections were then washed in PBS and mounted with ProLong Gold Antifade agent with or without DAPI (Life Technologies).

### Terminal Deoxynucleotidyl Transferase (TdT) dUTP Nick-End Labeling (TUNEL) Assay

Apoptotic cells in the spleen were detected by TUNEL assay following the manufacturer’s protocol (Roche Diagnostics, Mannheim, Germany) and imaged using a Zeiss Axiovert 200 microscope with ApoTome imaging and AxioVision 4.6 software (Zeiss).

### *In Situ* Enzyme Chemistry

After removal, kidney slices were snap frozen immediately in liquid nitrogen. The tissues were cryosectioned (6 μm thick) and stained for COX activity, as described previously (Lebrecht et al., 2004).

### Electron Microscopy

Freshly collected kidney tissues (∼2 mm thick) were fixed in 2.5% glutaraldehyde and 4% paraformaldehyde for 48 h and were processed for electron microscopy, as previously described (Waters et al., 2004).

### Cell Culture

The mIMCD-3 cell line (ATCC) is a polarized inner medullary collecting duct epithelial cell line derived from a mouse transgenic for the early region of SV40 [Tg(SV40E)bri/7] (Rauchman et al., 1993). Cultures were maintained in ATCC recommended DMEM:F-12 medium containing 5% FBS, 100 U/ml penicillin, and 100 µg/ml streptomycin. Cells (1e4 to 1e5) were grown in 24-, 48-well plates or chamber slides (10,000/well) and cultured at 37°C in a humidified atmosphere of 5% CO_2_ to a density of 80% confluence. Experiments were performed on cells from passages 2–5.

### Collection of Serum From LPS Treated Mice and Treatment of Cells

Male, 10-week-old mice were injected with PBS, or Hamp (50 µg/mouse, i.p.) and 24 h later were injected with 6.5 mg/kg LPS (i.p.) and euthanized 4 h later. Serum from four to five mice in respective experimental groups (PBS, PBS+ LPS, or LPS+ hemcidin) was pooled and stored in −80°C till further use. Cultured IMCD3 cells were first treated with 1 µg/ml Hamp or PBS in 5% FBS for 8 h. Following Hamp treatment, the cells were cultured for another 8 h in 5% serum from mice treated with LPS for 4 h. In separate experiments, serum from PBS, LPS, or Hamp (−24 h) + LPS treated mice was collected 4 h after LPS treatment. mIMCD-3 cells were incubated with 5% serum from the three groups for 8 h. Cells and supernatants were analyzed for viability, iNOS generation, and lipid peroxidation.

### siRNA Transfection and H-ferritin Knockdown Studies

J774A.1 a mouse monocyte/macrophage cell line from ATCC was used for H-ferritin knockdown studies. This cell line has been used to study the effects of Hamp (Nevitt and Thiele, 2011). Cells were maintained in DMEM (high glucose) containing 10% FBS, 100 U/ml penicillin, and 100 lg/ml streptomycin; 80–90% confluent cells were pretreated with 10 µg/ml Hamp or PBS for 6 h and then exposed to 10 ng/ml LPS for 4 h and processed for RNA isolation. Silencer Select siRNA to H-ferritin and negative control no. 1 siRNA (catalog number 4390771 and AM4611, respectively) were purchased from Thermo Fisher Scientific. About 2 × 10^5^ macrophages were transfected for 24 h using GenMute siRNA transfection reagent and siRNA to H-ferritin or negative control siRNA as per manufacturer’s instructions. After the transfection period, cells were grown in 0.5% medium containing 10 μg/ml Hamp or PBS for 6 h. Following this, cells were exposed to 5 ng/ml LPS for 4 h and culture supernatant was used for analysis.

### Western Blot Analysis

Snap-frozen tissue sections/cells were homogenized in Tris-Triton tissue lysis buffer containing complete protease inhibitor cocktail (Halt Protease and Phosphatase Inhibitor Cocktail. Thermo Scientific). Protein content in the homogenate was estimated using the Pierce BCA Protein Estimation Kit (Thermo Fisher Scientific, Rockford, IL). Ferroportin was measured in membrane fractions isolated from the whole spleen lysate using a membrane isolation kit (Thermo Fisher Scientific). H-ferritin was measured in whole spleen lysates. Twenty to thirty micrograms of protein per sample was loaded on a 10% NuPAGE Bis-Tris gel. The resolved proteins were transferred onto a nitrocellulose membrane (LI-COR Biotechnology, Lincoln, NE). Equal protein loading was confirmed using Ponceau staining for ferroportin and glyceraldehyde 3-phosphate dehydrogenase (GAPDH) for H-ferritin. Samples were probed with rabbit anti-mouse ferroportin (Novus Biologics) and goat anti-mouse H-ferritin (Santa Cruz) antibody and detected with donkey anti-rabbit Alexa 800 or donkey anti-goat Alexa 800 antibody (LI-COR) antibodies. Mouse monoclonal glyceraldehyde 3-phosphate dehydrogenase (GAPDH) (Abcam) was used as the loading control and detected with donkey anti-mouse Alexa 680 antibody (LI-COR). Blots were visualized on Odyssey CLx Imaging System (LI-COR). Quantitation of data was performed using densitometry software (Image Studio, LI-COR).

### Assay for Cell Viability

The percentage of lactate dehydrogenase (LDH) released from cells into the media was used as a measure of cell viability, using the LDH Cytotoxicity Assay Kit (Cayman Chemical, Ann Arbor, MI) as directed by the manufacturer. Data are expressed as the percentage of total cellular LDH released by the cells.

### Flow Cytometry

mIMCD-3 cells were trypsinized at the end of incubation times, washed two times with buffer (PBS containing 5% bovine serum albumin) to remove trypsin. The cells were then incubated with anti-CD16/32 (Fc block, clone 2.4g2; eBioscience, San Diego, CA) and fixed/permeabilized with BD Cytofix/Cytoperm as per manufacturer’s instructions. Following fixation/permeabilization, cells were stained with PE-conjugated iNOS (N-20) antibody (Santa Cruz). Flow cytometry data were acquired using BD FACSCalibur (BD Biosciences, San Jose, CA) with Cytek eight-color flow cytometry upgrade (Cytek Development, Fremont, CA) and analyzed with FlowJo software 9.0 (Tree Star Inc., Ashland, OR); 200,000 events/sample were acquired. Doublets were neglected from analysis. Gating strategy is shown in **Supplemental Figure 2**.

### Fluorescence Microscopy for Detection of Reactive Oxygen Species

mIMCD3 cells grown in chamber slides were loaded BODIPY 581/591 C11, (Thermo Fisher) to detect reactive oxygen species (ROS) in cytoplasm and membrane. Oxidation of the polyunsaturated butadienyl portion of the dye results in a shift of the fluorescence emission peak from ∼590 to ∼510 nm. Cells were then washed with PBS to remove any free probe prior to treatment with DMEM:F-12 containing 5% pooled serum from PBS, PBS + LPS, or Hamp + LPS treated mice. After 6 h, cells were visualized on a Zeiss Axiovert 200 microscope with ApoTome imaging and AxioVision 4.6 software (Zeiss).

### Serum TNF-α, IL-6, IL-1β, and MCP-1 Measurement

Serum TNFα, IL-6, IL-1β, and MCP-1 levels were measured using commercial ELISA kits (eBioscience), as per manufacturer’s instructions.

### Serum Iron Parameters

Serum iron (colorimetric assay) and transferrin (immunologic assay) and transferrin saturation were measured at the University of Virginia’s clinical laboratory facilities.

### Real-Time PCR

RNA isolation and cDNA synthesis was carried out as described previously (Scindia et al., 2015). Briefly, tissue was lysed using Qiagen Tissue Lyser and RNA was extracted using the RNeasy Plus Mini Kit (Qiagen, Hilden, Germany) as per manufacturer’s instructions. Predesigned primers for NGAL, KIM-1, TNFα, IL-6, IL-23a, PGC-1α, IL-22, and endothelin were purchased from Bio-Rad. Beta actin was amplified in parallel and used as the reference gene. Data are expressed as fold change over control and were calculated using either the 2^−ΔC(T)^ or 2^−ΔΔC(T)^ method.

### Statistics

Statistical significance was determined using two-tailed unpaired Student’s t-test. P < 0.05 at 95% confidence interval was considered significant. Mann–Whitney test was used for samples not passing normality test. One-way or two-way analysis of variance (ANOVA) was used to compare more than two groups of experimental conditions. All the analysis were performed using GraphPad Prism 6 (GraphPad Inc).

## Results

### Hamp Pretreatment Reduces Serum Iron and Protects Against LPS-Induced AKI

**Figure 1:**
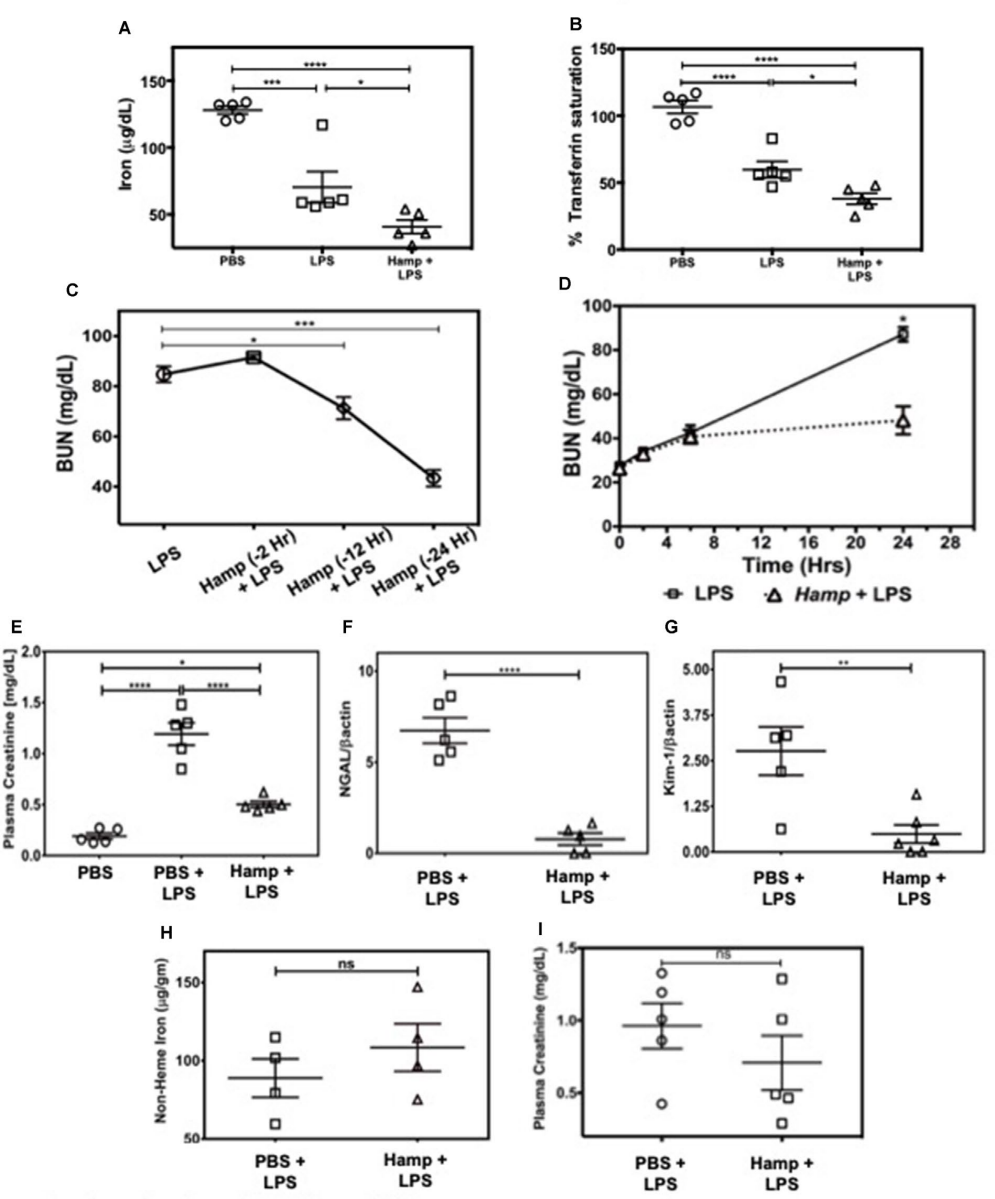
Hamp pretreatment reduces LPS-induced AKI. Mice were injected Hamp (50 µg/mouse, I.P.) 24 hrs before receiving 6.5 mg/kg LPS and euthanized 24 hr later to measure serum iron and transferrin saturation. Compared to PBS, LPS administration significantly decreased serum iron and transferrin saturation, both of which were further significantly reduced by Hamp pretreatment **(A-B)**. Hamp (50 µg/mouse, I.P.) was administered 2, 12 or 24 hr before LPS (6.5 mg/kg). BUN was measured 24 hours later **(C)**. Mice ptretreated with PBS or Hamp 24 hours before LPS administration were tail bled at 0, 2, 6 and 24 hours to measure changes in BUN **(D)**. Hamp pretreatment protected the kidneys and reduced LPS-induced increase in plasma creatinine **(E)**, NGAL **(F)**, and KIM-1 **(G)** gene expression. Non-heme iron was measured following acid digestion of kidney **(H)**, normalized to tissue weight and expressed as micrograms per gram of tissue. Hamp treatment did not significantly reduce plasma creatinine, an indicator of renal function when injected 3 hrs after LPS **(I)**. *P < 0.05, **P < 0.005, ***P < 0.0005, ****P < 0.0001. Data points are plotted as mean ± SEM (n = 4-5 per group). Experiments were repeated twice and representative data from a single experiment is depicted.

First, we measured the changes in serum iron and transferrin saturation (Tsat) following PBS, LPS, or Hamp + LPS injections (i.p. Hamp, 50 µg). The dose and timing of Hamp injection were based on previous studies by Rivera et al. (2005), who demonstrated that 50 µg Hamp lowers serum iron for more than 48 h after injection. This corresponds to 1.4 µM Hamp in the serum and is consistent with the inhibitory concentration of 50% (IC50) of hepcidin measured *in vitro*. Serum iron in PBS treated mice was 171 ± 11 µg/dl, which was reduced to 28 ± 1 µg/dl following 24 h of hepcidin pretreatment. However, the hemoglobin content was normal (13.3 ± 1.3 g/dl PBS vs. 13.6 ± 1.5 g/dl Hamp). Thus, mice were low on circulating iron but had normal hemoglobin levels after hepcidin treatment prior to induction of endotoxemia. Hamp by itself did not induce any significant renal injury as measured by plasma creatinine, BUN, NGAL, and KIM-1 (**Supplementary Figure 1A–D**). Compared to PBS, LPS significantly reduced serum iron and Tsat (**Figure 1A and B**), both of which were further significantly lowered by Hamp pretreatment. Next, we injected C57BL/6 (WT) mice intraperitoneally (i.p.) with Hamp or PBS, at different time points (**Figure 1C**) before LPS administration (6.5 mg/kg) and measured AKI. Compared to PBS, Hamp (50 µg) given 12 h before LPS significantly reduced BUN (**Figure 1C**) but was most effective when administered 24 h before LPS (**Figure 1C**). LPS caused an early and mild AKI (high BUN) in both groups during the first 6 h, which progressively worsened in PBS group, but was arrested and significantly better at 24 h in the Hamp-treated mice **(** **Figure 1D** **)**. Plasma creatinine values correlated with the BUN data at 24 h **(** **Figure 1E** **)**. Renal gene expression of tubular injury markers, NGAL, and kidney injury molecule-1 (KIM-1) were also significantly reduced in Hamp-treated mice (**Figure 1F and G**). Interestingly, renal non-heme iron content, 24 h following LPS injection in both PBS and Hamp treated mice (given Hamp 24 h before LPS), was comparable (**Figure 1H**). Hamp (50 µg) given 3 h post LPS resulted in a non-significant decrease in plasma creatinine (**Figure 1I**).

### Hamp Pretreatment Reduces LPS-Induced TNFα and Immune Cell Infiltration and Is Associated With Preserved Renal Ultrastructure and Mitochondria

**Figure 2:**
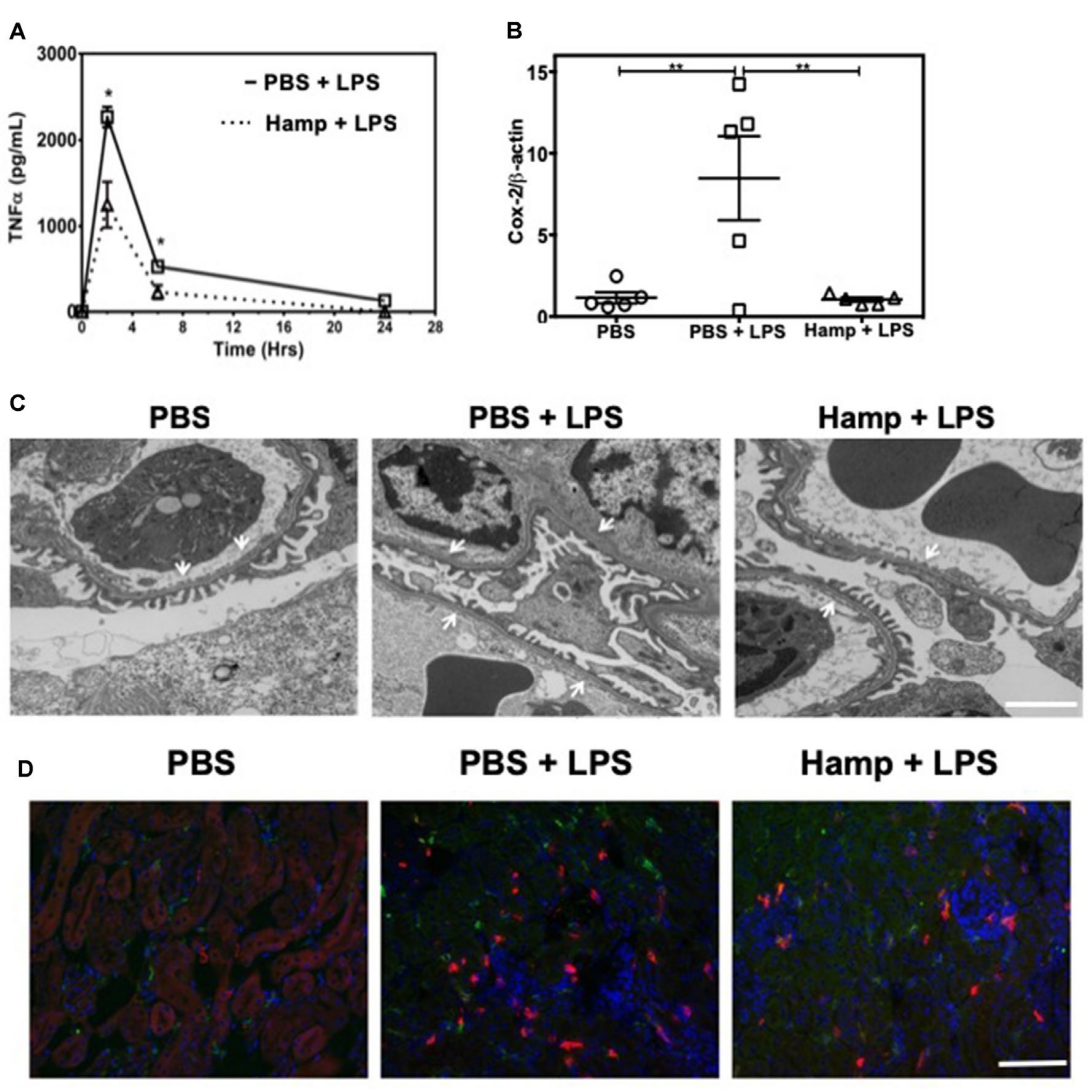
Hamp reduces LPS-induce systemic TNFα, and mediators of inflammation like Cox-2 and protects the renal ultra-structure and reduces immune cell infiltration. Mice were treated with PBS or Hamp (50 µg/mouse, I.P.) 24 hr before injecting them with 6.5 mg/kg LPS. Hamp pretreatment significantly reduced LPS-induced serum TNFα levels at 2 and 6 hours **(A)**. Similarly renal Cox-2 gene expression was also reduced by hepcidin pretreatment **(B)**. *P < 0.05, **P < 0.01. Data points are plotted as mean ± SEM (n = 4-5 per group). Representative electron micrographs of glomeruli from three groups of mice are shown. PBS treated mice show a glomerular capillary with normal fenestrated endothelium and podocyte foot processes which are fused following LPS show a glomerular capillary with normal fenestrated endothelium and podocyte foot processes which are fused following LPS treatment. **(C)**. This loss/fusion of the endothelial fenestrae is reduced by Hamp pretreatment. **(C)**. Original Magnification: 8,000X, Scale bar = 2 µm. Following LPS treament, there were a large number of neutrophils (7/4 antigen; magenta), CD11b+ve cells (CD11b; red) and macrophages (F4/80: green) in PBS-treated mice that were reduced by Hamp treatment **(D)**. Scale bar = 100 µm. Experiments with 4-5 mice each were repeated twice and representative data from a singe experiment is depicted.

TNFα is a rapidly released cytokine implicated in the pathogenesis of LPS-induced AKI (Cunningham et al., 2002; Lin and Yeh, 2005; Zarjou and Agarwal, 2011). We measured systemic TNFα levels at different time points after LPS injection in both PBS and Hamp-pretreated mice. Two hours after LPS injection, PBS group showed a sharp increase in serum TNFα, which decreased rapidly by 6 h (**Figure 2A**). Hamp pretreatment significantly reduced LPS-induced TNFα at both these time points (**Figure 2A**). LPS-induced upregulation of Cox-2 gene was also significantly reduced by Hamp pretreatment (**Figure 2B**). The increase in systemic TNFα and Cox-2 were associated with glomerular ultrastructural changes. Compared to PBS, electron micrographs of PBS + LPS group showed a loss and fusion of glomerular endothelial fenestrae (**Figure 2C**). This feature was absent in Hamp + LPS group. The damage to the renal ultra-structure was associated with infiltration of 7/4^+^ neutrophils, CD11b^+^ cells, and F4/80^+^ macrophages in the PBS group, which was reduced by Hamp pretreatment (**Figure 2D**). We did not observe obvious renal tubular damage by histology in any of the groups. It is known that even in severe sepsis, AKI can develop in the absence of overt histological or immunohistological changes and may be functional in nature (Langenberg et al., 2014). LPS-induced endotoxemia affects renal mitochondrial function (Tran et al., 2011) and reduces PPARγ coactivator-1α (PGC-1α), the master regulator of mitochondrial biogenesis and metabolism. Compared to PBS, LPS treatment significantly reduced PGC-1α gene expression, which was significantly attenuated by Hamp pretreatment (**Supplemental Figure 2A**). LPS-induced loss of PGC-1α was associated with mitochondrial dysfunction, as indicated by *in situ* activity of electron transport chain enzyme complex cytochrome c oxidase (COX) (**Supplemental Figure 2B**). Decreased PGC-1α and COX activity correlated with ultrastructural changes in renal mitochondria, which were swollen and had rarefied cristae in LPS-treated mice and this was not observed in Hamp + LPS treated mice (**Supplemental Figure 2C**).

### Hamp Protects Against LPS-Induced AKI by Reducing Systemic Inflammation and Not by Directly Acting on Renal Parenchymal Cells

**Figure 3:**
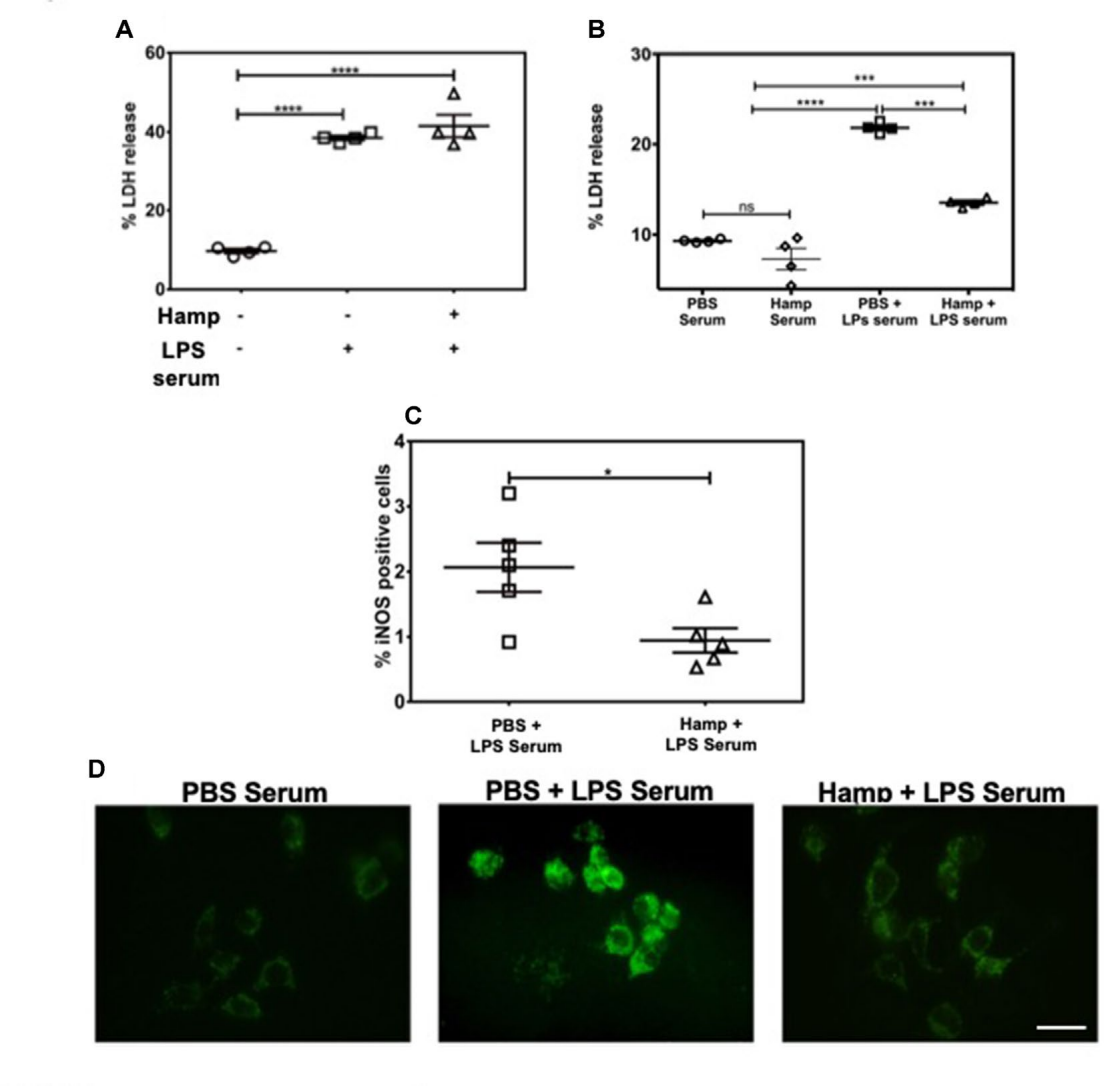
Hamp protects renal cells by reducing LPS-induced serum toxicity. mIMCD-3 cells (1e5) were grown in 5% FBS containing PBS or Hamp (1 µg/ml, 8 hr) and then incubated with 5% serum from LPS treated mice for 8 h. Cells treated with either PBS or Hamp and exposed to serum from LPS treated mice released similar and significantly more LDH compared to cells exposed to serum from PBS treated mice **(A)**. 5% serum from PBS + LPS treated mice induced a significantly higher LDH release from mIMCD-3 cells (1e5) compared to serum from PBS only and Hamp + LPS treated mice **(B)**. Compared to 5% PBS + LPS serum, Hamp + LPS serum significantly reduced iNOS generation in mIMCD-3 cell **(C)**. *P < 0.05, ***P < 0.0005, ****P < 0.0001. Data points are plotted as mean ± SEM. Green fluorenscence intensity of BODIPY 581/591 C11 was used to identify and localize ROS **(D)**. Oxidation of the polyunsaturated butadienyl portion of the dye results in a shift of the fluorescence emission peak from ∼590 nm to ∼510 nm. Compared to cells incubated with serum from PBS and Hamp + LPS treated mice, FITC fluorescence was highest and localized to both surface and intracellular compartments in a punctate in PBS + LPS serum–treated cells **(D)**. Experiments were repeated twice and representative data from a single experiment is depicted.

LPS induces a rapid increase in serum cytokines (Erickson and Banks, 2011). The combination of these cytokines and other serum factors can be toxic to the renal parenchyma. Hamp directly protects the renal tubular cells against hemoglobin-induced AKI (Van Swelm et al., 2016). In order to evaluate Hamp’s direct cytoprotective effect in the context of sepsis, we chose mouse inner medullary collecting duct cells (mIMCD-3) which have been previously used to study mechanisms of sepsis-induced injury (Pathak and Mayeux, 2010). Serum from mice treated with 6.5 mg/kg LPS for 4 h was used as the cytotoxic serum. mIMCD-3 cells were pretreated for 8 h with PBS or Hamp (1µg/mL), followed by 5% cytotoxic serum for another 8 h. The culture supernatants were analyzed for LDH release. The mIMCD-3 cells incubated with serum of PBS-treated mice were used as controls and released a basal level of LDH. Cytotoxicity was significantly and comparably increased by serum from LPS-treated mice in both PBS and Hamp pretreated mIMCD-3 cells (**Figure 3A**). Similarly, mouse glomerular endothelial cells pretreated with 1 µg/ml Hamp or PBS for 8 h followed by 1 µg/ml LPS for 14 h showed similar gene expression of endothelin (**Supplemental Figure 3**). Both these observations suggest that Hamp does not directly protect the renal parenchymal cells against LPS or toxic serum from LPS-treated mice.

We next asked whether systemic Hamp pretreatment reduces cytotoxic ability of serum in LPS-treated mice. For this purpose, supernatants of mIMCD-3 cells were analyzed for LDH release after 8 h incubation with 5% serum from either PBS, LPS, or Hamp + LPS-treated mice (mice were given Hamp 24 h before injecting 6.5 mg/kg LPS and serum was collected 4 h later). Compared to serum from PBS-treated mice, cells incubated with serum of LPS-treated mice released significantly greater amount of LDH, which was significantly reduced in the group treated with serum from Hamp + LPS-treated mice (**Figure 3B**). Next, we investigated the mechanism of this cytotoxicity using inducible nitric oxide synthase (iNOS) and ROS generation in mIMCD-3 cells. After 8 h, cells treated with serum from LPS-treated mice were iNOS positive (detected by flow cytometry, 2.066 ± 0.377). This was significantly reduced in cells treated with serum from Hamp + LPS mice (0.943 ± 0.187) (**Figure 3C** **,** **Supplementary Figure 4**). Higher iNOS levels in the cells treated with serum from LPS-treated mice resulted in increased intracellular ROS and lipid peroxidation as indicated by greater fluorescence shift of BODIPY C11 labeled IMCD3 cells, which was in the order LPS serum > Hamp + LPS serum > PBS serum (**Figure 3D**). To further highlight the importance of systemic inflammation in reducing endotoxin-induced AKI, we compared renal injury in naive and SPN-X mice, 24 h after LPS (6.5 mg/kg) injection (**Supplemental Figure 5A**). Though studies have investigated role of spleen in the pathogenesis of sepsis (Hiraoka et al., 1995; Suzuki et al., 1996; Shih-Ching et al., 2004), it is unknown if SPN-X would protect against sepsis-induced AKI, and whether spleen is required for hepcidin to mediate its protection. Compared to naive mice, SPN-X significantly reduced early release of TNFα, measured 2 h post LPS injection (SPN-X mice: 1,051 ± 95.59, **Supplemental Figure 5B** Vs naive mice: 2,266 ± 118, **Figure 2A**), and this was further lowered by Hamp pretreatment (886.5 ± 85.55) (**Supplemental Figure 5B**). The reduction in inflammation following SPN-X was associated with significantly lower plasma creatinine, renal NGAL and KIM-1 gene expression in both PBS and Hamp-treated mice (**Supplemental Figure 5C–E**).

### Hamp Treatment Is Associated With Increased Anti-Inflammatory Molecule H-ferritin

**Figure 4:**
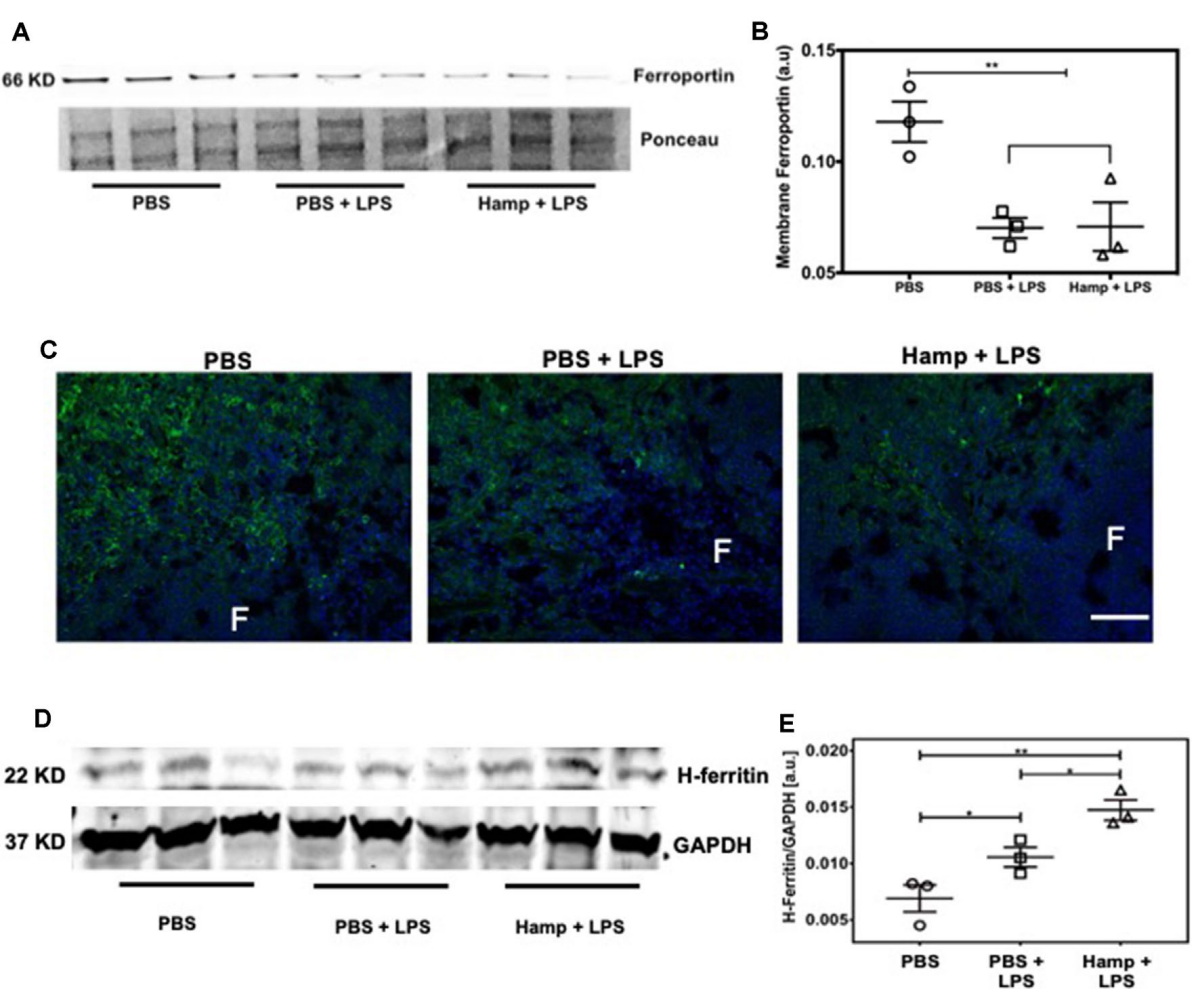
Hamp treatment is associated with increased anti-inflammatory molecule H-ferritin. Mice were treated with PBS or Hamp (50 µg/mouse, I.P.) 24 hr before injecting them with 6.5 mg/kg LPS. Quantitative western blotting of spleens showed that both LPS and Hamp + LPS treatment significantly and similarly reduced Ferroportin **(A-B)**. Immunofluorescence labelling of spleen membrane fraction showed expression of ferroportin (green) in red pulp region of PBS treated mice **(C, first panel)**. However, ferroportin was decreased in both PBS + LPS and Hamp + LPS treated spleens and could be detected in only a few scattered cells in the red pulp region **(C, middle panel)**. "F": Splenic B cell follicle. Expression of H-ferritin in whole spleen lysates was measured by quantitative western blots. GAPDH was used to confirm equal protein loading and normalize H-ferritin. Compared to PBS, LPS treatment significantly increasesd splenic H-ferritin expression, which in turn was significantly increased in Hamp + LPS treated mice **(D-E)**. *P < 0.05, **P < 0.005. Experiments (n = 4-5) were repeated twice and representative data from a single experiment is depicted.

Previous studies have shown that both LPS and Hamp reduce ferroportin expression (Liu et al., 2005; Viatte et al., 2005; Scindia et al., 2015). We observed significant drop in serum iron following LPS treatment, which was further reduced in Hamp + LPS treated mice (**Figure 1A and B**), suggesting further reduction in ferroportin-mediated iron export in Hamp-treated mice. Hamp decreases ferroportin expression rapidly on splenic iron-cycling red pulp macrophages, also one of the primary sites of systemic iron retention (Chung et al., 2009); therefore, we measured splenic ferroportin expression by both quantitative Western blotting and immunofluorescence after PBS, PBS + LPS, and Hamp + LPS treatment. Compared to PBS, mice treated with both PBS + LPS and Hamp + LPS had a significantly lower but comparable expression of ferroportin (**Figure 4A and B**), which was mostly observed in the splenic red pulp (**Figure 4C**). Ferroportin downregulation is expected to increase intracellular iron and result in a concomitant increase of H-ferritin through an iron-regulatory response. Quantitative Western blotting indicated that LPS treatment significantly increased H-ferritin expression compared to PBS treatment and the expression was further higher in Hamp + LPS group (**Figure 4D and E**).

### Hamp Pretreatment Reduces the LPS-Induced Splenic Inflammatory Signature

**Figure 5:**
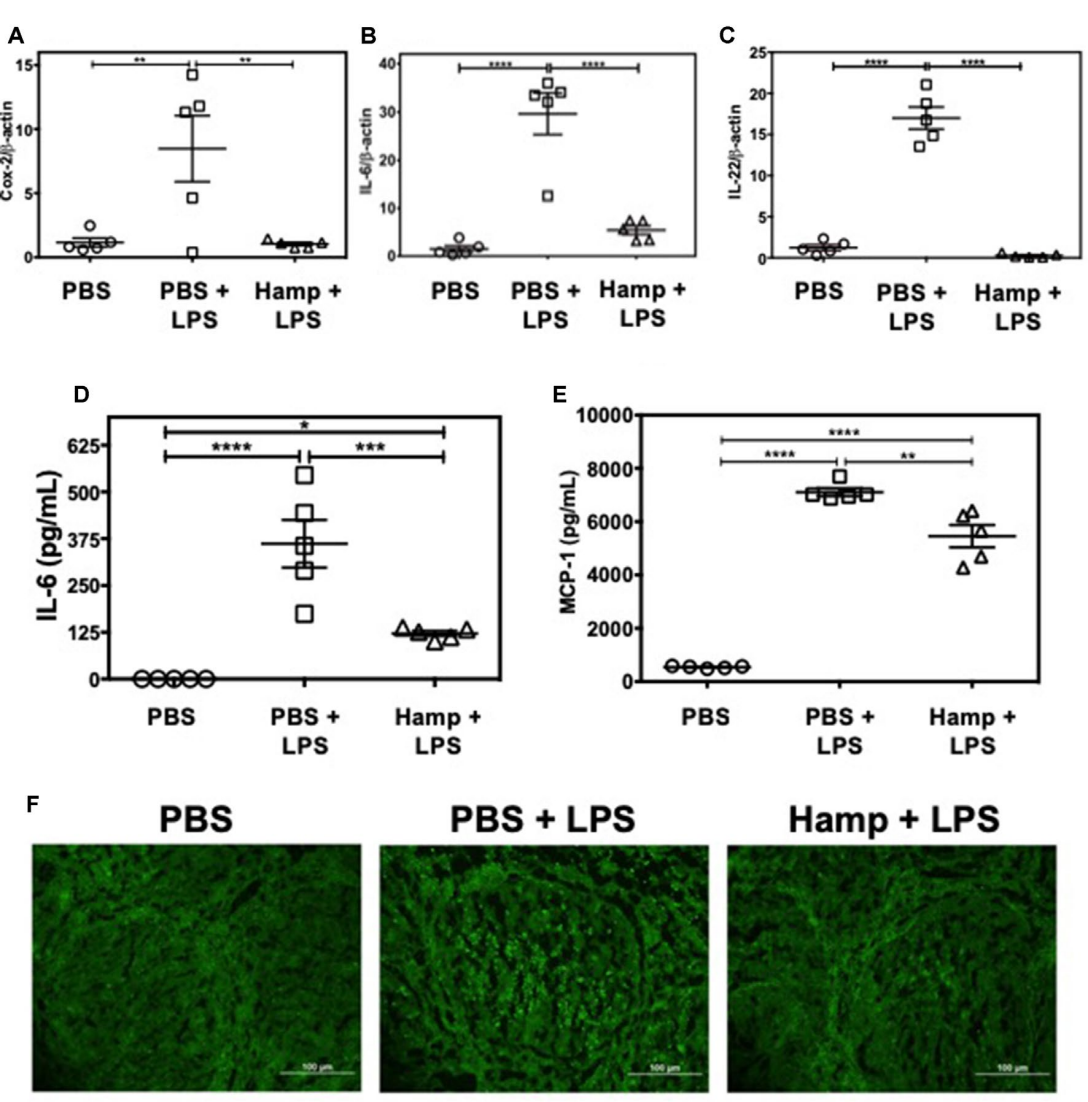
Hamp pretreatment reduces splenic inflammatory signature following LPS-induced SIRS. Mice were treated with PBS or Hamp (50 µg/mouse, I.P.) 24 hr before injecting them with 6.5 mg/kg LPS. Hamp pretreatment reduced LPS-induced upregulation of splenic Cox-2, IL-6, and IL-22 genes **(A-C)**. Similarly, LPS-induced increase in serum IL-6 and MCP-1 were significantly reduced by Hamp pretreatment **(D-E)**. *P < 0.05, **P < 0.005, ***P < 0.0005, ****P < 0.0001. Data points are plotted as mean ± SEM (n = 4-5 per group). TUNEL reactivity (Green) was used to assay splenic cell apoptosis after LPS administration. PBS treated mice did not show signs of apoptosis **(F; first panel)**. PBS + LPS treated mice showed several FITC positive, apoptotic cells in the splenic follicle **(F; middle panel)**, which was markedly reduced in the Hamp + LPS treated mice **(F; last panel)**. Representative images from two different experiments with 4-5 mice each are depicted.

LPS triggers rapid cytokine production and activation-induced lymphocyte apoptosis in the spleen and thymus (Honda et al., 2016; Liu et al., 2016). We observed that compared to PBS, PBS + LPS group had significantly higher splenic gene expression of Cox-2, IL-6, and IL-22. These were significantly reduced by Hamp pretreatment (**Figure 5A–C**). Similarly, LPS significantly increased serum IL-6 and MCP-1 levels, which was significantly reduced by Hamp pretreatment (**Figure 5D and E**). The LPS-induced increase in cytokines was associated with increase in TUNEL positive cells within the splenic follicles, and this too was reduced by Hamp pretreatment (**Figure 5F**).

### Hamp’s Ability to Mitigate LPS-Induced Inflammatory Response is Macrophage H-ferritin Dependent

**Figure 6:**
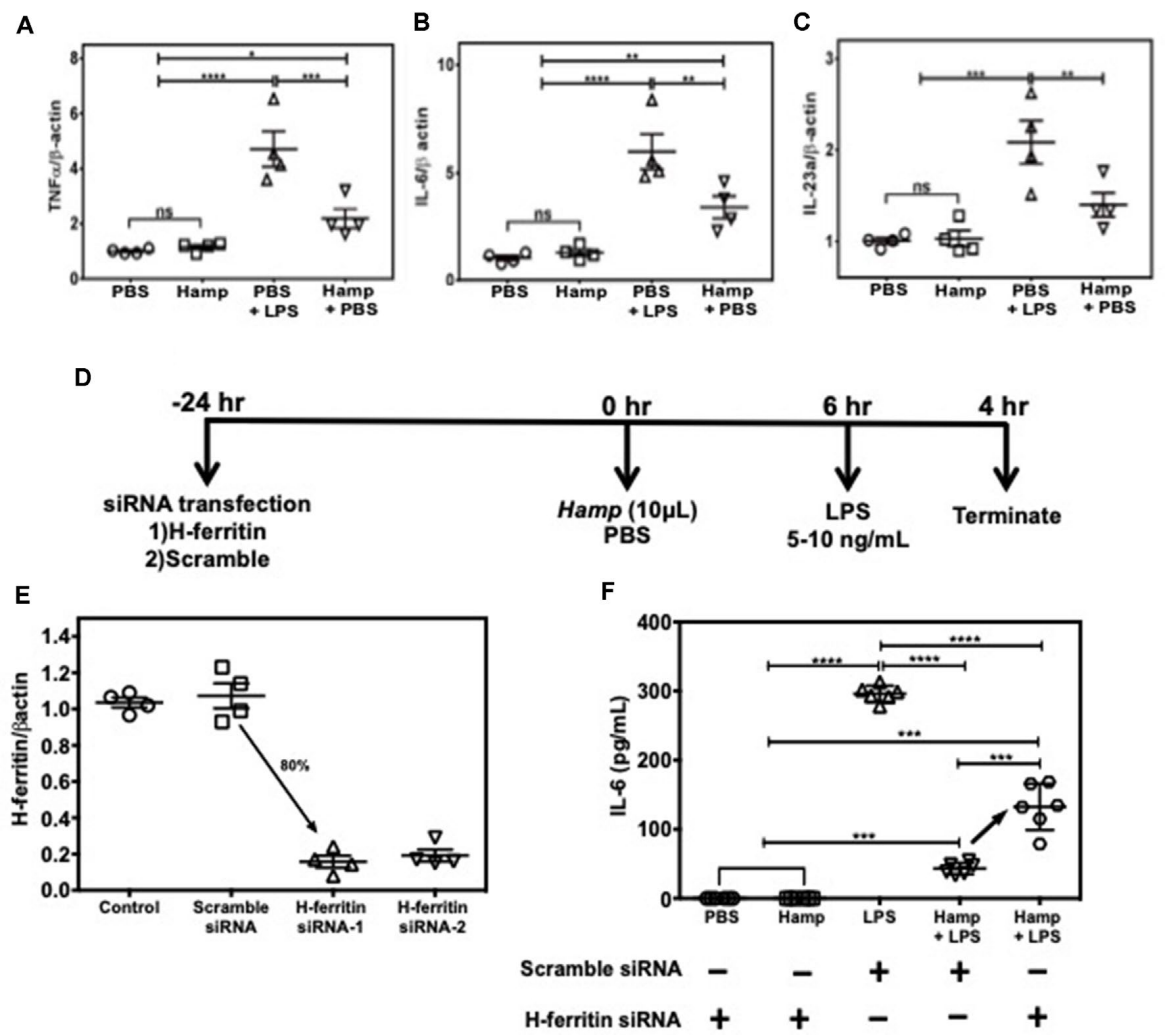
Hamp’s ability to mitigate LPS-induced inflammatory response in macrophages is H-ferritin dependent. 90% confluent J774A.1 cells were pretreated with 10 µg/mL Hamp or PBS for 6 hours and then exposed to 10 ng/mL LPS for 4 hrs. Hamp by itself did not induce an inflammatory response in cultured macrophages. As expected, LPS significantly increased expression of TNFα, IL-6, IL-23a, all three of which were significantly reduced by pretreatment with Hamp **(A, B, C)**. Study design for H-ferritin knockdown in macrophages **(D)**. 2.5e5 J774A.1 cells were treated with siRNA to H-ferritin resulted in ∼ 80% knockdown of H-ferritin gene **(E)**. Following knockdown, cells were treated with PBS or Hamp (10 µg/mL) and 5 ng/mL LPS and the supernatants were analyzed for IL-6. H-ferritin siRNA treated cells incubated with PBS or Hamp did not elicit an IL-6 response **(F)**. In scramble siRNA treated cells, LPS significantly increased IL-6 levels, which was significantly reduced by Hamp pretretment **(F)**. Compared to cells treated with scramble siRNA, H-ferritin knockdown cells treated with Hamp + LPS secreted significantly higher quantities of IL-6 **(F)**. *P < 0.05, **P < 0.005, ***P < 0.0005, ****P < 0.0001. Experiments were repeated twice with 4-6 experiment wells per conditions and representative data is shown.

Among various immune cell populations, Hamp is primarily known to target macrophages, the primary storage cell for iron in the body. Therefore, we examined Hamp’s ability to directly modulate immune response in macrophages using J774a cells, an established mouse macrophage cell line. LPS-induced pro-inflammatory genes like TNFα, IL-6, and IL-23a were significantly reduced in cells pretreated with Hamp (**Figure 6A–C**). As Hamp pretreatment was associated with highest splenic H-ferritin levels and reduced inflammation following LPS administration (**Figures 4** and **5**), we evaluated the role of H-ferritin in Hamp’s ability to protect against LPS-induced inflammation. We measured the response of H-ferritin knockdown J774A cells to LPS following PBS or Hamp treatment. Knockdown of H-ferritin (∼80%) was achieved using siRNA (**Figure 6D and E**). Incubation with PBS or Hamp did not elicit detectable IL-6 response in H-ferritin knockdown or scramble siRNA treated cells. Hamp significantly reduced LPS-induced increased IL-6 in scramble siRNA treated cells (**Figure 6F**). This ability of Hamp to reduce LPS-induced IL-6 was significantly reduced in H-ferritin-deficient cells (**Figure 6F**). These results clearly indicate that H-ferritin is critical for Hamp-induced mitigation of LPS-induced inflammation.

### Hamp Reduces Polymicrobial Sepsis Induced AKI and Mortality

**Figure 7:**
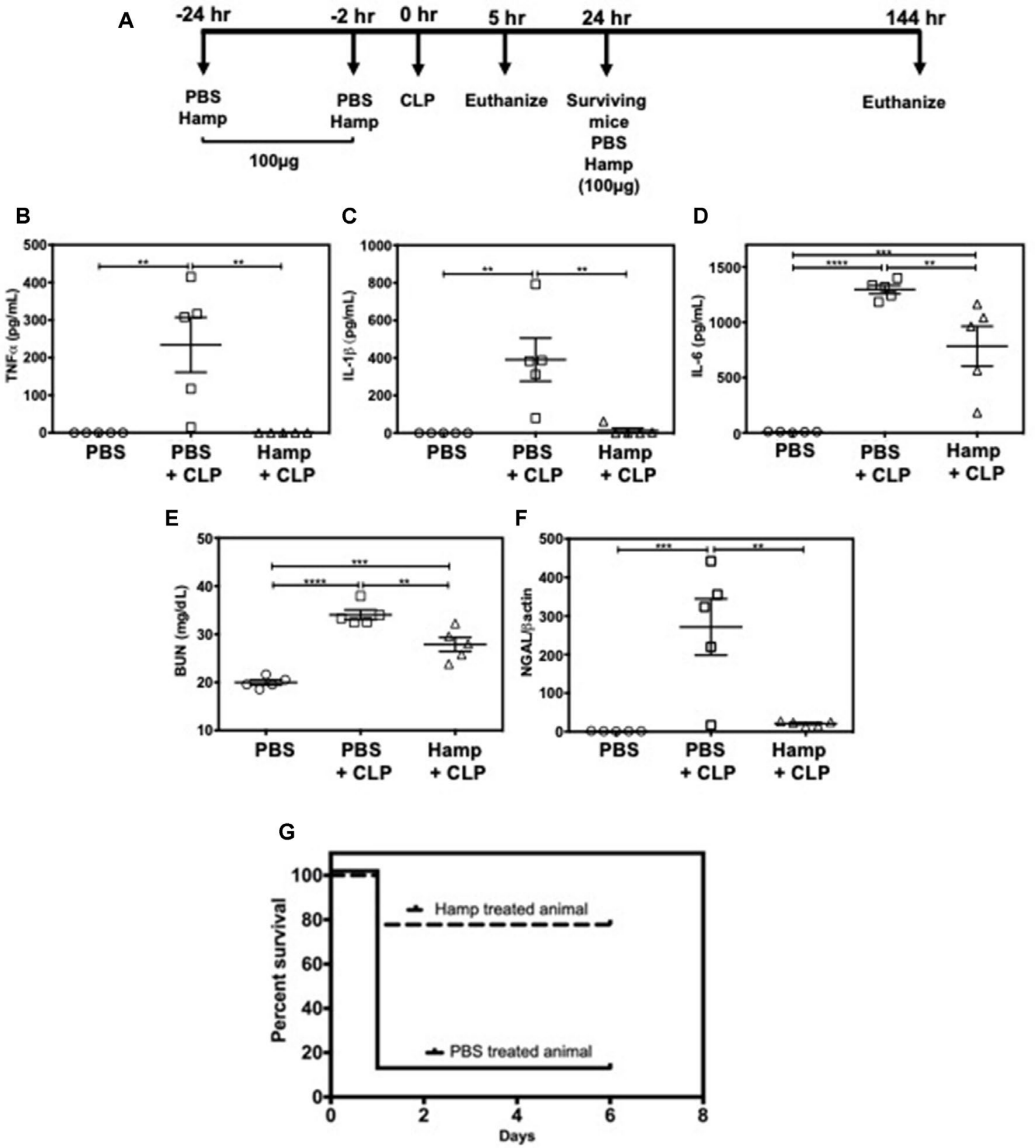
Hamp treatment reduces CLP-induced AKI and mortality. Study design and treatment strategy for CLP experiments **(A)**. Hamp treatment (100 µg/mouse, I.P., as indicated in **Figure 7A** reduced CLP-induced increase in serum TNFα, IL-6, five hours post CLP surgery **(B–D)**. At this time point. AKI as measured by BUN (renal function) **(E)** and renal NGAL gene expression (renal tubular injury) **(F)** were significantly lower in the Hamp treated mice. *P < 0.05, **P < 0.005, ***P < 0.0005, ****P < 0.0001. Data points are plotted as mean ± SEM (n = 5 per group). Survival curves show an improved survival in Hamp-treated mice compared to PBS-treated ones following CLP surgery **(G)**. Data are plotted as Kaplan-Meier survival curve. By 24 hrs, there was 90% mortality in PBS treated mice subjected to CLP surgery, whereas 80% of Hamp treated mice survived for up to 6 days’ post-surgery. Experiments (n = 5) were repeated twice and representative data from a single experiment is depicted.

We also established the protective role of Hamp in settings of polymicrobial sepsis induced by cecal ligation and puncture (CLP). Compared to sham operated mice, PBS-treated mice had significantly higher levels of TNFα, IL-1β, and IL-6, 5 h after CLP. All CLP-induced cytokines were significantly reduced by prophylactic Hamp treatment (**Figure 7A–D**). Further, Hamp treatment was associated with a better-preserved renal function (BUN), reduced renal tubular injury (as measured by renal NGAL gene expression) (**Figure 7E and F**). CLP-induced inflammation and renal injury in PBS group was associated with 90% mortality by 24 h, whereas only 20% of Hamp-treated mice died at this time and survived for up to 6 days (**Figure 7G**).

### Therapeutic Benefit of Hamp in Polymicrobial Sepsis Induced Bacteremia and AKI

**Figure 8:**
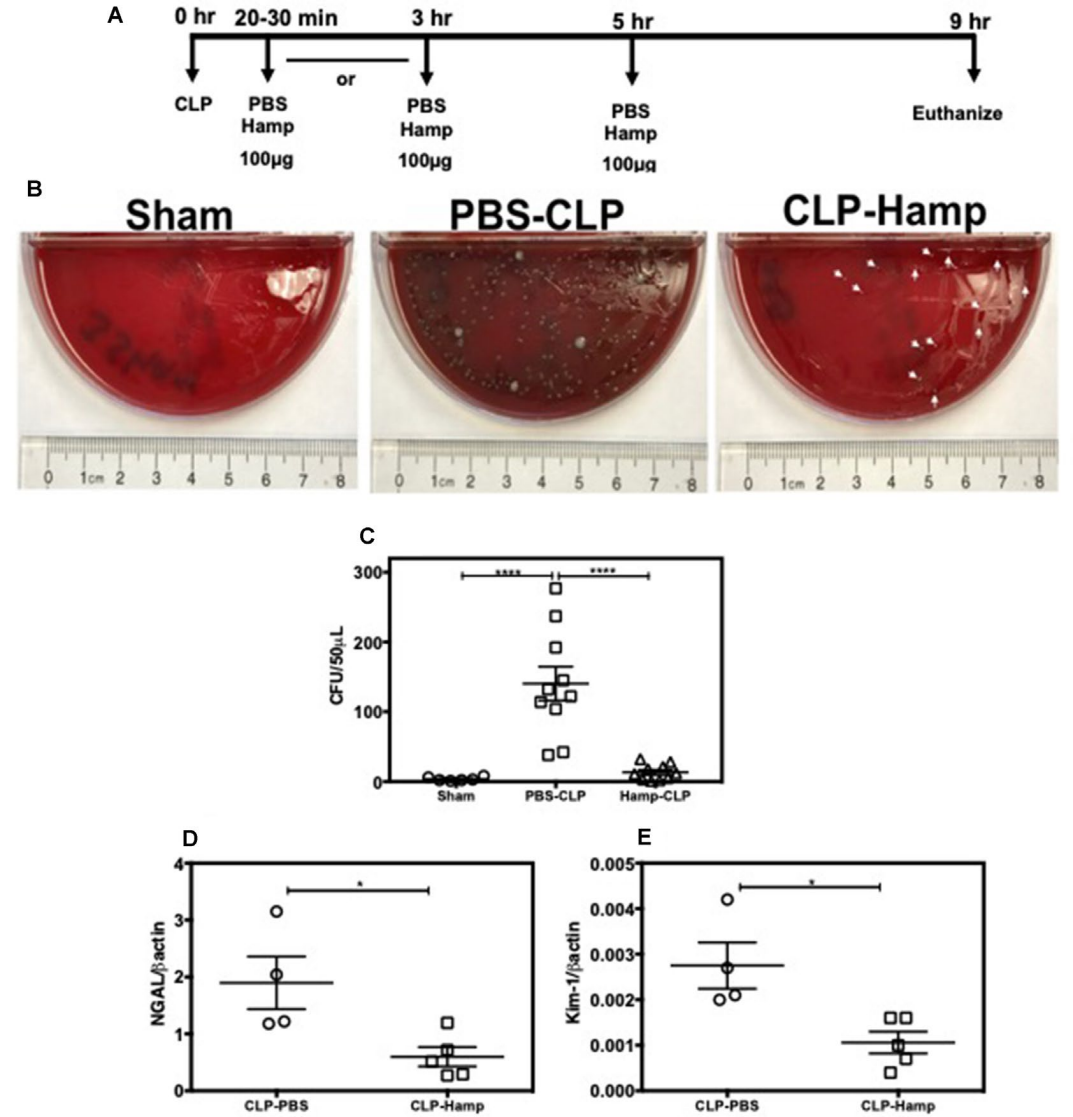
Hamp reduces bacteremia and AKI when administered after the onset of sepsis. Study design and therapeutic treatment of Hamp in CLP settings **(A)**. Hamp injection (100µg/mouse, I.P., as indicated in **Figure 8A** within 20-30 mins post CLP, followed by another dose 5 hrs later significantly reduced bacteremia in mice **(B-C)**. Kidney tubular injury as measured by renal gene expression of NGAL **(D)** and KIM-1 **(E)** was also significantly reduced in this group. *P < 0.05, ***P < 0.0005. Data points are plotted as mean ± SEM (n = 4-5 per group). Experiments were repeated twice and representative data from a single experiment is depicted.

We evaluated the therapeutic potential of Hepcidin when administered after the onset of polymicrobial sepsis induced by CLP. Mice were subjected to either sham surgery or CLP and were given Hamp 20–30 min or 3 h later. Second dose of Hamp was administered 5 h later and tissue was harvested 9 h after the surgery (**Figure 8A**). Mice administered Hamp (100 µg) 3 h after CLP had comparable AKI (as indicated by BUN values) to PBS treated mice by 9 h (43.54 ± 8.51 CLP + PBS vs 48 ± 4.83 CLP + HAMP). However, mice that received Hamp therapy within 30 min of CLP displayed significantly lower bacteremia (**Figure 8B and C**) and AKI (as measured by renal NGAL and KIM gene expression) (**Figure 8D and E**).

## Discussion

In this study, we demonstrate the prophylactic and therapeutic effect of Hamp in mitigating sepsis-associated AKI. Notably, Hamp pretreatment reduced LPS- and peritonitis-induced systemic inflammation, splenocyte apoptosis, AKI, and mortality. Functionally, Hamp pretreatment was associated with preserved renal function and glomerular ultrastructure following LPS administration and this was independent of renal iron content. With *in vitro* studies, we provide evidence that Hamp does not directly protect renal parenchyma against SA-AKI, instead it decreases systemic cytotoxic milieu that result in reduced renal tubular epithelial cell death. Taken along with our data from SPN-X mice, these findings establish an important role of systemic inflammation in the pathogenesis of endotoxin-induced AKI. Our *in vitro* studies demonstrate the mechanistic importance of macrophage H-ferritin in mediating Hamp’s anti-inflammatory effect. Of further clinical relevance, we demonstrate the therapeutic benefit of Hamp administered after the onset of polymicrobial sepsis in controlling bacteremia and mitigating AKI.

The growth of pathogens in their hosts is critically dependent on the pathogens’ ability to capture and utilize iron. Hamp-induced hypoferremia has been proposed as an important host defense mechanism (Ganz, 2009; Drakesmith and Prentice, 2012). Thus, ability of Hamp to further decrease serum iron and increase tissue H-ferritin following LPS administration (**Figure 1**) may benefit the host by limiting iron availability to the invading pathogens.

Studies by Zager et al. (2005) have shown that intravenous iron potentiates LPS-induced TNFα release. The increase in TNFα and TNFR1 lead to loss of glomerular endothelial cell fenestrae and damage the glomerular endothelial surface layer (Xu et al., 2014), with only mild histologic changes in both human and animal kidneys (Doi et al., 2009). The ability of Hamp to rapidly decrease serum iron is well established (Rivera et al., 2005) and Hamp levels increase following endotoxic insult (Armitage et al., 2011; Rodriguez et al., 2014). Thus, by iron sequestration and H-ferritin induction, Hamp reduces systemic TNFα levels and may mitigate TNFα-mediated renal injury. Pagani et al. (2011) showed that iron-deficient macrophages mount a stronger inflammatory response to LPS, and this could be dampened with Hamp treatment. Our *in vivo* findings support these conclusions. LPS also causes direct damage to the tubular mitochondria (Tran et al., 2011; Quoilin et al., 2014), which is accentuated by TNFα (Mariappan et al., 2007, Mariappan et al., 2009). We found that Hamp treatment was associated with reduced damage to the mitochondria (**Supplemental Figure 1**), likely to be primarily mediated through its anti-inflammatory cytokine reducing effects.

Serum toxicity experiments better mimic the complex milieu to which renal tubular cells are exposed to during endotoxemia (Pathak and Mayeux, 2010). Other *in vitro* models of renal tubular injury have used high concentrations of LPS and/or individual cytokines like IFN-γ and TNF-α (Markewitz et al., 1993; Du et al., 2006; Tiwari et al., 2006), thereby limiting the relevance to the complex tubular epithelial milieu during sepsis. Based on our studies on mIMCD-3 and MGEC cell lines, we can reasonably conclude that Hamp does not have a clinically relevant direct protective effect on these renal parenchymal cells from LPS or cytotoxic serum from LPS-treated mice. In contrast, systemic cytotoxic milieu following endotoxemia is significantly reduced by systemic Hamp pretreatment (**Figure 3**). The importance of early systemic inflammatory response in cytotoxicity and AKI is further validated by findings from our SPN-X data. In naive mice, LPS induced a sharp increase in TNFα within 2 h of injection (2,266 ± 118 pg/ml, **Figure 2A**), whereas progression to AKI (as measured by BUN) was observed only by 6 h (**Figure 1D**). At the similar time point, Hamp reduced LPS-induced TNFα (1,373 ± 223 pg/ml, **Figure 2A**) and this was associated with an arrest in progression to AKI. Similarly, SPL-X reduced serum TNFα, 2 h post LPS administration (1,051 ± 95.59, **Supplemental Figure 5B**) and this was associated with reduced AKI with no additional protection from exogenous Hamp. Thus, our observations suggest that strategies reducing early and systemic inflammatory response (Hepcidin or SPLN-X) are of more relevance than local mechanisms in mitigating endotoxin-induced AKI. Immune cells comprise more than 90% of the spleen and produce both TNFα and IL-6 in response to LPS (Suzuki et al., 1996; Honda et al., 2016). Macrophages are one of the major early sources of TNFα, IL-6, and prostaglandins during sepsis (Eliopoulos et al., 2002). As Hamp pretreatment further reduced TNFα (but without further improvement in AKI) in LPS treated SPN-X mice, we anticipate an extra-splenic anti-inflammatory effect of hepcidin. TLR4 expressed by hepatocytes regulates hepcidin expression following LPS stimulation (Lee et al., 2017); however, effect of Hamp on LPS stimulated hepatocytes is not known. To the best of our knowledge, there are no current methods to selectively deplete splenic iron-cycling macrophages without compromising other tissue-resident macrophages to tease out the role of splenic versus non-splenic immune cells.

Both LPS and Hamp cause internalization of ferroportin (Nemeth et al., 2004; Viatte et al., 2005) and splenic iron retention. We observed that LPS caused internalization of ferroportin in the splenic red pulp and a significant increase in splenic H-ferritin. While TNFα is known to cause apoptosis, it also induces iron-independent increase in cytoprotective H-ferritin (Miller et al., 1991; Cozzi et al., 2003). Overexpression of H-ferritin induces resistance from hydrogen peroxide toxicity (Cozzi et al., 2000). Our data show that Hamp + LPS treated mice have the highest H-ferritin levels and least splenic inflammation and apoptosis. Additive increase in H-ferritin in Hamp + LPS-treated mice despite similar levels of ferroportin downregulation suggests a functional (inhibitory) effect of hepcidin on ferroportin-mediated iron export. This correlated well with serum iron, which was significantly lower in the Hamp + LPS group compared to LPS-only group. Splenocyte apoptosis has been shown to be relevant in human sepsis and treatments that target immune cell apoptosis improve survival (Hotchkiss et al., 2001) We have previously reported that Hamp causes splenic iron retention and H-ferritin induction under settings of ischemic AKI (sterile AKI) and is associated with reduced inflammation (Scindia et al., 2015). Taken along with previous published studies (Cozzi et al., 2000, Cozzi et al., 2003), our current observations suggest that Hamp-mediated increase in splenic iron and H-ferritin protects spleen through reduction in splenic inflammation and apoptosis.

One of the consequences of Hamp treatment is intracellular iron retention and buildup of H-ferritin (Nemeth et al., 2004; Viatte et al., 2005). Our data with J774a macrophages show that Hamp reduces LPS-induced pro-inflammatory cytokines (**Figure 6**). Mechanistically, we provide novel evidence that Hamp-induced H-ferritin is critical for protection against LPS-mediated pathology. Unlike H-ferritin sufficient macrophages, we observed that its deficiency reduced effectiveness of Hamp to curtail inflammation (**Figure 6**). Mechanistic relevance of these findings is supported by recent studies showing that H-ferritin in hepatocytes and myeloid cells is critical for tolerance to sepsis (Weis et al., 2017; Weis S). H-ferritin is also upregulated by NF-κB in an iron-independent manner and inhibits TNFα-induced apoptosis by suppressing reactive oxygen species (Cozzi et al., 2000, Cozzi et al., 2003; Pham et al., 2004). Systemic TNF-α levels increase within 2 h of LPS injection, whereas discerning AKI sets in only by 6–8 h (**Figures 1B** and **2A**). Based on this known sequence of events, we hypothesize that Hamp increases H-ferritin in macrophages and dampens their immediate response to LPS. This is associated with reduced splenocyte apoptosis and cytokine profile, which collectively mitigate AKI.

**Figure 9:**
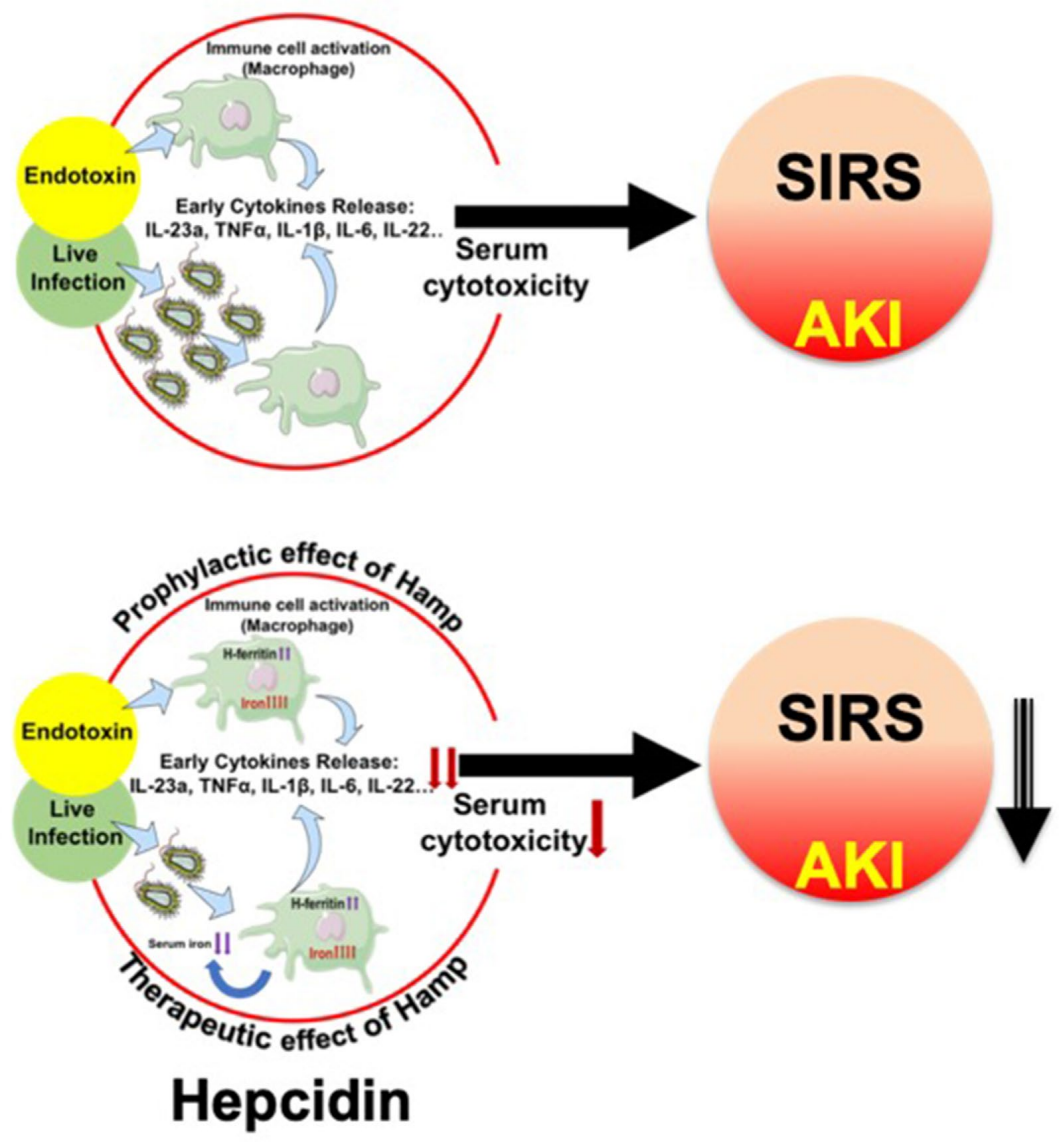
Proposed mechanism depicting hepcidin’s protective effect in settings of endotoxemia and live infections. Endotoxemia and live infections cause a rapid, systematic activation of immune cells, including macrophages, which secrete cytokines like TNFα, IL-6 and IL-1β. This toxic milieu initiates SIRS and eventually worsens AKI. Hepcidin pretreatment attenuates macrophage inflammatory response to endotoxin. In parallel, by sequestering iron within tissue (mostly macrophages), hepcidin limits iron availability, a key nutrient for invading pathogens and thereby reduces bacterial load. Thus, hepcidin limits AKI by dual mechanisms: 1) by reducing macrophage inflammatory response and reducing overall serum cytotoxicity, and 2) by reducing pathogen load.

Our data from the peritonitis model further establish the prophylactic and therapeutic benefit of Hamp against SA-AKI and mortality. De Domenico et al. (2010) have previously demonstrated the protective effect of prophylactically administered Hamp in CLP-induced mortality but any effect on bacteremia or AKI was not explored. Further, the therapeutic benefit of Hamp administered after the onset of sepsis was not examined in this study. In another recent study, Hamp deficiency was shown to increase bacteremia and decrease survival of following *Vibrio vulnificus* infection. This was partially ameliorated by dietary iron depletion and by timely administration of hepcidin agonists (Arezes et al., 2015). However, sepsis is a polymicrobial event (Hyde et al., 1990) involving both gram positive and negative bacteria. Our findings demonstrating the therapeutic benefit of Hamp when administered after the onset of polymicrobial sepsis and ensuing AKI are novel. **Figure 9** broadly describes the potential mechanism by which hepcidin protects against SIRS/peritonitis-induced AKI.

Hamp protects against SA-AKI or mortality only when administered within 30 min of CLP. Although this is a narrow window, it opens up the consideration to use Hamp as a therapy immediately after the onset of severe sepsis. For example, the prevalence rates of polymicrobial bacteremia are high in cleft lip surgery, cleft palate surgery, and alveoloplasty (40.9%, 33.3%, and 50%, respectively) (Adeyemo et al., 2013). Prophylactic administration as a preventative approach or early therapy with Hamp may also benefit at-risk or high-risk surgical patients with increase in AKI biomarkers from developing focal or systemic infection, sepsis, and overt AKI. There are other indications to consider such as after intra-operative bowel perforation, or in bowel ischemia in abdominal vascular surgery.

Sepsis is biphasic event involving an hyper and hypo-inflammatory state (Hotchkiss et al., 2013a, Hotchkiss et al., 2013b), and sustained immunosuppression worsens outcomes. However, the effect of Hamp is transient (Rivera et al., 2005) which is advantageous as can attenuate the early hyper-inflammatory response, without compromising the ability to fight late or recurrent infection. Furthermore, unlike other anti-inflammatory therapies that have been tested, Hamp also reduces bacteremia, which is advantageous in sepsis, particularly in an era of emerging antimicrobial resistance where identifying novel therapies are crucial.

Taken together, our data in two clinically relevant mouse model of sepsis-induced AKI suggest that Hamp regulation may play a key role in protection against kidney injury in sepsis. Hamp targets iron homeostasis and protects, both when administered prophylactically or after the onset of sepsis. Synthetic human Hamp is being tested in a phase 2 clinical trial for the treatment of iron overload in adult patients with hereditary hemochromatosis. This highlights the translational potential of Hamp in septic patients. In the era of emerging antimicrobial resistance, our study suggests a therapeutic potential for hepcidin in human sepsis, sepsis-associated inflammation, and AKI.

## Ethics Statement

All experiments were performed in accordance with the National Institutes of Health and Institutional Animal Care and Use Guidelines. The Animal Care and Use Committee of the University of Virginia approved all procedures and protocols.

## Author Contributions

YS and SS conceived and YS, EW, and SS designed the study. YS and EW performed most of the experiments. VL, JLee, SC, EG, and JLed assisted with flow cytometry, Western blots, and RNA isolation. YS, EW and SS analyzed and interpreted most of the data. All authors read and edited the manuscript. YS and SS wrote the final version of the manuscript.

## Funding

Research reported in this manuscript was supported by *the National Institute of Diabetes and Digestive and Kidney Disease* of the National Institutes of Health under award number R01DK103043 to SS.

## Conflict of Interest Statement

The authors declare that the research was conducted in the absence of any commercial or financial relationships that could be construed as a potential conflict of interest.
