# Supplementary material for: Protective Role of Hepcidin in Polymicrobial Sepsis and Acute Kidney Injury
Source: Front Pharmacol. 2019 Jun 6;10:615. doi: 10.3389/fphar.2019.00615 (PMC6563000; doi:10.3389/fphar.2019.00615)
Supplement: Supplementary file 1 [file DataSheet_1.pdf]

Supplemental Figure 1

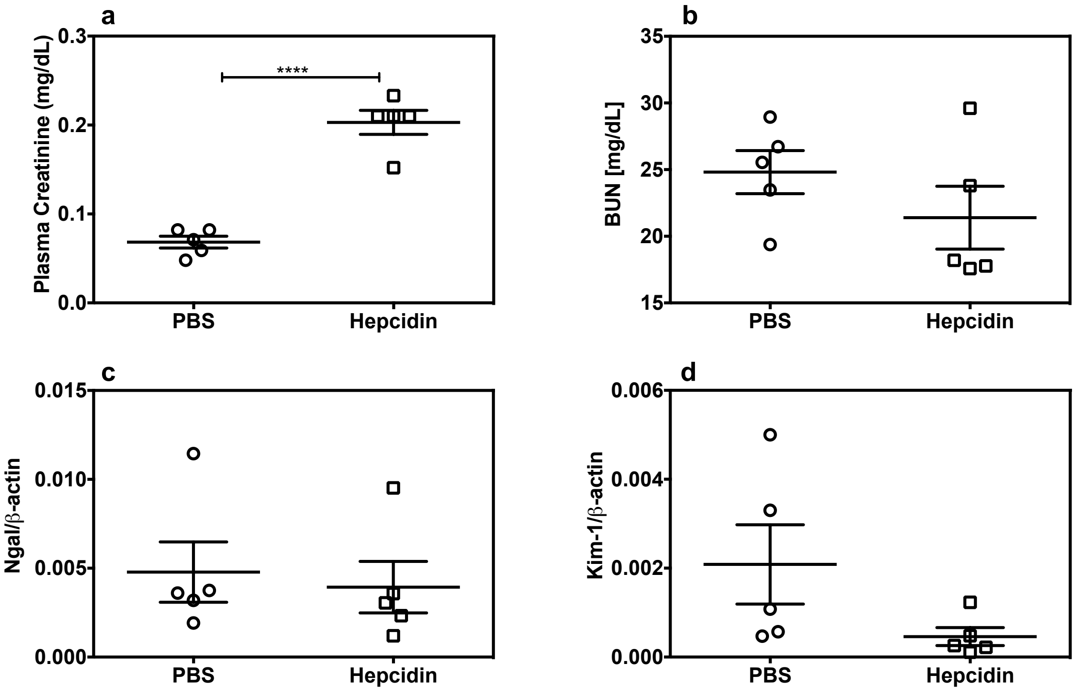

Hamp does not cause AKI in naïve, uninfected mice.

Mice were treated with PBS or Hamp (50μg/mouse, I.P.) and euthanized 24 hrs later. Though there was a slight yet significant increase in plasma creatinine following hepcidin treatment, it was less than 0.3 mg/dL (did not meet criteria for AKI) compared to PBS injected mice and the values were low **(a)**. BUN (b), renal NGAL gene(c) and renal KIM-1 gene expression were comparable between PBS and Hepcidin treated mice. ***P < 0.0005. Data points are plotted as mean ± SEM. Experiments (n = 4-5) were repeated twice and representative data from a single experiment is depicted.

Supplemental Figure 2

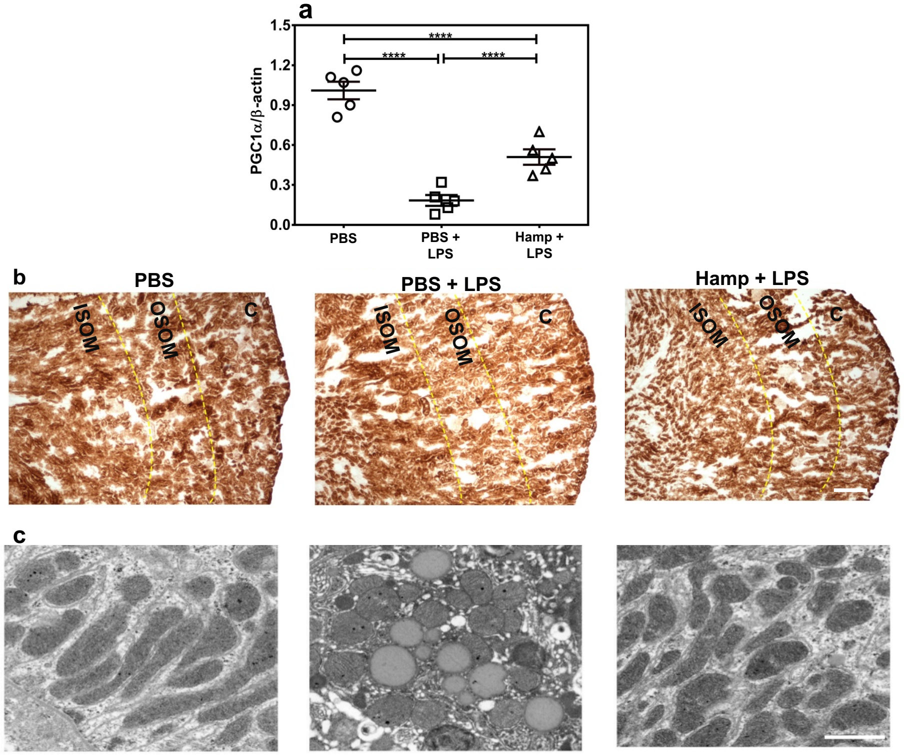

Hamp mitigates LPS-induced mitochondrial dysfunction and injury.

Mice were treated with PBS or Hamp (50μg/mouse, I.P.) 24 hr before injecting them with 6.5 mg/kg LPS. Prior Hamp treatment reduced LPS-induced decrease in gene expression of renal PGC-1α **(a)**. ***P < 0.0005, ****P < 0.0001. Data points are plotted as mean ± SEM. Staining (brown) for cytochrome *c* oxidase enzyme activity on snap-frozen kidneys 24 hours after PBS, PBS + LPS or Hamp + LPS treatment. Cortex (C) and inner stripe of the outer medulla (ISOM) have the most intense staining, followed by outer stripe of the outer medulla (OSOM). The enzymatic activity is greatly reduced following LPS treatment in the cortex and OSOM. These changes are reduced by pretreatment with Hamp. Scale bar = 50 μm **(b)**. Transmission EM of renal tubules demonstrates swelling of mitochondria and rarefaction of cristae after LPS treatment, both of which are reduced by Hamp pretreatment **(c)**. Original Magnification: 12,000X, Scale bar = 1 μm. Experiments (*n* = 4-5) were repeated twice and representative data from a single experiment is depicted.

Supplemental Figure 3.

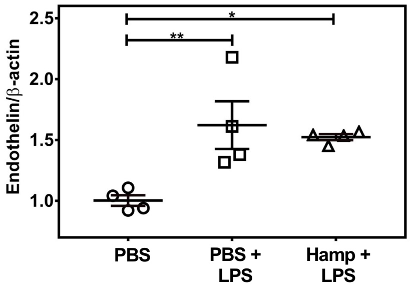

Primary mouse glomerular endothelial cells (MGEC) from C57BL/6 mice (Cell Biologics) were maintained in complete endothelial cell medium provided by the supplier. Cells were initially plated in T-25 flask, cultured at 37^{0}C in a humidified atmosphere of 5% CO2 to a density of 80% confluence and expanded for 2 passages. The primary cells were then split into 48 well plates. Cells were grown overnight in 0.5% medium, and treated with 1µg/mL of hepcidin for 8 hrs. Following hepcidin treatment, cells were to exposed to media containing 1µg/mL LPS for 14 hrs. At the end of incubation period cells were washed and RNA was extracted for gene expression studies.

Compared to PBS treated cells, LPS significantly increased endothelin gene expression in MGEC. Pretreatment with hepcidin did not reduce LPS-induced endothelin expression. *P < 0.05, **P < 0.005. Data points are plotted as mean ± SEM (n = 4 per group). Experiments were repeated twice and data from a representative data from an individual experiment is shown.

Supplemental Figure 4.

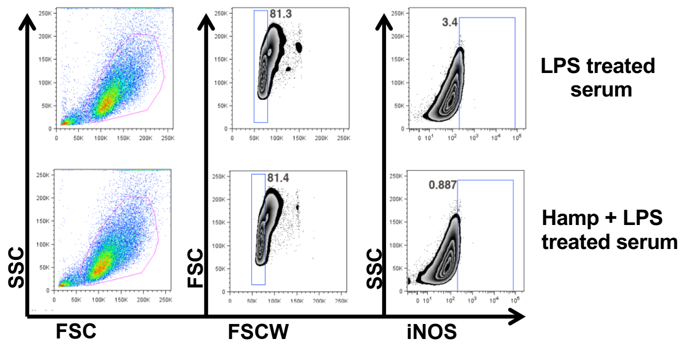

Gating strategy for mIMCD-3 cells incubated with 5% pooled serum from PBS + LPS or Hepcidin + LPS treated mice.

Supplemental Figure 5

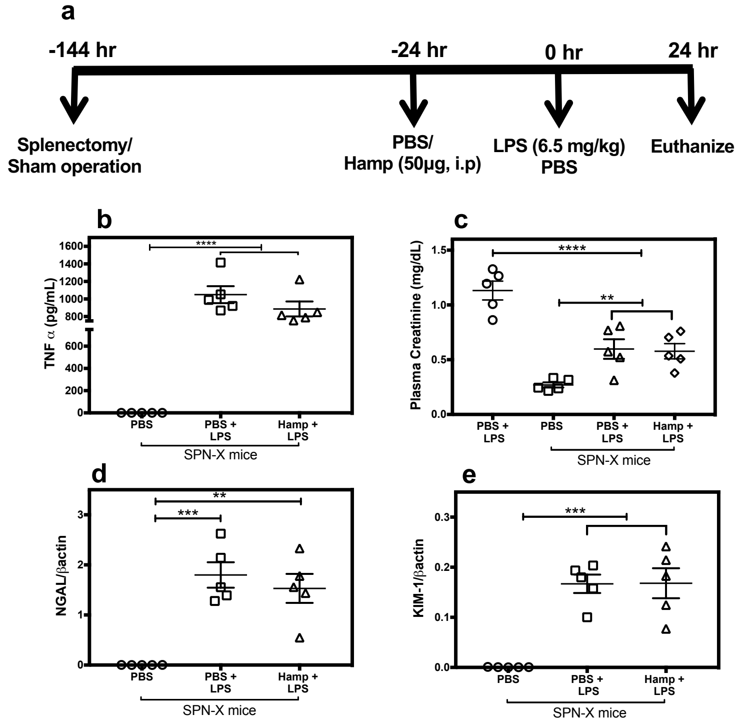

Splenectomy (SPN-X) protects against LPS-induced AKI independent of exogenous Hamp.

Seven-week-old splenectomized mice were purchased from Jackson laboratory and used for experiments one week later. Mice were injected with either Hamp (50µg) or PBS followed by 6.5 mg/kg LPS, 24 hrs later. Study design and treatment strategy for SPN-X experiments **(a)**. Hamp pretreatment reduced serum TNFα in LPS injected SPN-X mice, suggesting extra splenic anti-inflammatory effects **(b).** SPN-X by itself did not induce any renal injury. Compared to sham operated mice, SPN-X mice injected with LPS had significantly preserved renal function (lower AKI) as indicated by plasma creatinine **(c)**. Hamp pretreatment of SPN-X mice did not further improve renal function following LPS injection **(c)**. Renal tubular injury in SPN-X mice was reduced following LPS injection as indicated by low expression of NGAL **(d)** and KIM-1 **(e)**. $*P < 0.05$, $**P < 0.005$, $***P < 0.0005$, $****P < 0.0001$. Data points are plotted as mean ± SEM (n = 5 per group). Experiments (n = 5) were repeated twice and representative data from a single experiment is depicted.
